# Effects of fetal growth restriction on the perinatal neurovascular unit and possible treatment targets

**DOI:** 10.1038/s41390-023-02805-w

**Published:** 2023-09-06

**Authors:** Bing Anthony Wu, Kirat K. Chand, Alexander Bell, Suzanne L. Miller, Paul B. Colditz, Atul Malhotra, Julie A. Wixey

**Affiliations:** 1 Department of Paediatrics, Monash University, Melbourne, VIC Australia; 2 UQ Centre for Clinical Research, Faculty of Medicine, The University of Queensland, Brisbane, QLD Australia; 3 Department of Obstetrics and Gynaecology, Monash University, Melbourne, VIC Australia; 4 The Ritchie Centre, Hudson Institute of Medical Research, Melbourne, VIC Australia; 5 Perinatal Research Centre, Royal Brisbane and Women’s Hospital, Brisbane, QLD Australia; 6 Monash Newborn, Monash Children’s Hospital, Melbourne, VIC Australia

## Abstract

**Abstract:**

The neurovascular unit (NVU) within the brain is a multicellular unit that synergistically acts to maintain blood–brain barrier function and meet cerebral metabolic demand. Recent studies have indicated disruption to the NVU is associated with neuropathology in the perinatal brain. Infants with fetal growth restriction (FGR) are known to be at increased risk of neurodevelopmental conditions including motor, learning, and behavioural deficits. There are currently no neuroprotective treatments for these conditions. In this review, we analyse large animal studies examining the effects of FGR on the perinatal NVU. These studies show altered vascularity in the FGR brain as well as blood–brain barrier dysfunction due to underlying cellular changes, mediated by neuroinflammation. Neuroinflammation is a key mechanism associated with pathological effects in the FGR brain. Hence, targeting inflammation may be key to preserving the multicellular NVU and providing neuroprotection in FGR. A number of maternal and postnatal therapies with anti-inflammatory components have been investigated in FGR animal models examining targets for amelioration of NVU disruption. Each therapy showed promise by uniquely ameliorating the adverse effects of FGR on multiple aspects of the NVU. The successful implementation of a clinically viable neuroprotective treatment has the potential to improve outcomes for neonates affected by FGR.

**Impact:**

- Disruption to the neurovascular unit is associated with neuropathology in fetal growth restriction.
- Inflammation is a key mechanism associated with neurovascular unit disruption in the growth-restricted brain.
- Anti-inflammatory treatments ameliorate adverse effects on the neurovascular unit and may provide neuroprotection.

## Introduction

The neurovascular unit (NVU) is a multicellular unit within the brain consisting of neurons, perivascular astrocytes, microglia, pericytes, endothelial cells (ECs), and the basement membrane (BM).^1^ The NVU primarily functions to maintain the integrity of the blood–brain barrier (BBB) and meet cerebral metabolic demand. It was formally conceptualised in 2001, after which the neuroscience community began to make significant strides in NVU research.^2^ Research into the developing perinatal NVU, however, remains in its infancy.^2^ A handful of studies have now examined the effects of common perinatal insults on the NVU.^1^

Fetal growth restriction (FGR) is a common pregnancy complication affecting 5–10% of pregnancies,^3,4^ with higher rates in low-income countries.^5^ FGR is defined as a condition in which the fetus fails to reach its growth potential.^6^ FGR may occur due to maternal (nutrition, hypertension), placental, or fetal compromise (congenital, infection), but in most cases, the cause is placental insufficiency.^7,8^ Placental insufficiency results in inadequate transfer of oxygen and nutrients to the developing fetus, and subsequently causes chronic fetal hypoxia and hypoglycaemia.^9–11^ Fetal hypoxia induces an adaptive cardiovascular response that preferentially directs cardiac output to essential organs, including the brain, heart and adrenals.^5^ The change in the distribution of fetal circulation causes asymmetrical growth in the fetus and alters organ growth and development across multiple systems.^5^ In turn, FGR infants are at increased risk of various postnatal morbidities, the most significant being cardiovascular, pulmonary, and neurological.^12^

Neuropathologies associated with FGR are varied and complex in origin and include structural deficits such as reduction in brain volume and cellular losses.^5^ As a result, affected individuals are commonly subject to many short-term and long-term neurological impairments as well as functional deficits. FGR infants suffer worse motor and cognitive outcomes when compared to appropriate for gestational age (AGA) infants at 2 years of age as well as worse school-age outcomes.^13,14^ FGR infants, especially those born >32 weeks of gestation, are particularly vulnerable to intraventricular haemorrhage (IVH),^15,16^ and are at significantly higher risk of developing cerebral palsy.^17–20^

Most of the current literature reporting on the effects of FGR on the NVU are in large animal models. This is pertinent as even though small animal models of any disease state are important to study mechanisms of injury, the ability to use a large animal model may result in rapid translation of treatments to the clinic. This review discusses the effects of FGR on the perinatal NVU, and potential treatments to protect the FGR brain.

## The neurovascular unit

**Fig. 1:**
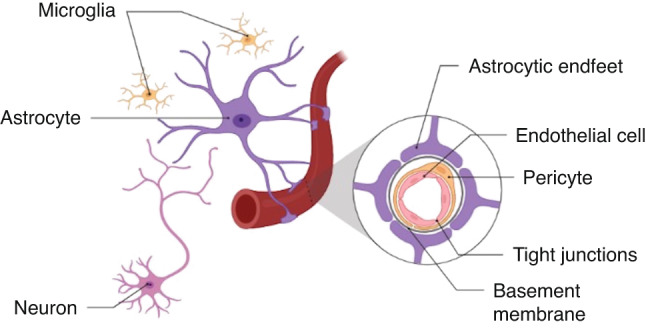
Structure of the neurovascular unit. Neurons regulate the NVU by communicating with astrocytes that surround the vasculature with specialised end feet. Pericytes can be found between the end feet and endothelial cells. Endothelial cells are held together through tight junctions to make up the vascular wall, acting as a physical barrier to maintain BBB function. Surrounding endothelial cells and pericytes is the continuous basement membrane, acting as a supporting structure for each component of the NVU.

A comprehensive review detailing the cellular components and function of the NVU in the perinatal brain was recently published.^1^ Each component has a unique role in contributing to the function of the NVU (Fig. 1).

### Components of NVU

Neurons act as the ‘pacemaker’ to regulate cerebral blood flow (CBF) via neurovascular coupling (NVC). In response to increased metabolic demand, neurons communicate with neighbouring astrocytes which influence intraluminal diameter of local cerebral vessels, via the release of vasoactive mediators, to enhance CBF. NVU astrocyte ‘end-feet’ projections wrap around to virtually cover all brain arterioles and capillaries.^21^ Astrocytes are key to initiating BBB formation and stability,^22^ as well as being involved in the recycling of neurotransmitters and ions via water channels and ion transporters.^23,24^ The BBB acts as the functional barrier of the NVU, critical to regulating the optimal brain environment.^25^

Pericytes, like the astrocytic end feet, are directly in contact with vasculature and play a vital role in the development of the BBB. Generally, the extent of pericyte coverage directly correlates with BBB integrity.^26^ It exerts its function by responding to neuron signals to regulate astrocytic end-feet attachment.^27^ Under the pericytes and astrocytes are ECs that line the vasculature and form the core anatomical unit of the BBB.^28^ ECs maintain BBB functionality passively through tight junctions (TJs) between adjacent cells, as well as actively through selective transport. Pericytes and ECs produce proteins that form an extracellular matrix called the BM,^29^ which provides essential structural support by acting as an anchoring structure for the above-mentioned surrounding cells.

### Function of NVU

The NVU has two primary functions—CBF regulation through NVC, and selective permeability of BBB.

NVC is also referred to as functional hyperaemia, which refers to the regulation of CBF at a microscopic level. Hyperaemia ensures that local regions in the brain receive oxygen and nutrients appropriate for its level of metabolic demand.^30^ This includes vasodilation in regions of the brain experiencing higher loads.^31^ This is facilitated by the components of the NVU where linking neurons are thought to release glutamate, activating nearby astrocytes and pericytes.^32^ These cells subsequently interact with cerebral vessels to adjust their intraluminal diameter, thereby regulating local CBF.^1^

The NVU plays a critical role in the formation and development of the BBB. ECs of the NVU contribute significantly to the selective permeability of the BBB through the formation of a physical barrier.^1^ This is done via inter-endothelial TJs, thereby restricting the paracellular movement of hydrophilic and large molecules into the central nervous system.^33,34^ This results in a BBB that is permeable only to small, lipophilic molecules, and carrier-mediated transport is required for important molecules such as glucose and neurotransmitters.^33,35^ In addition to ECs, other components of the NVU also play important roles in the maintenance of the BBB, most notably via pericyte and astrocyte end-feet coverage, whereby greater coverage is associated with stronger integrity and selective permeability of the BBB.^26,27,36,37^

## Effects of FGR on the perinatal NVU

The NVU plays a central role in adult neurodegenerative conditions.^38,39^ Although commonly regarded as immature and therefore more vulnerable than adult BBB, emerging evidence suggests the developing BBB has function equal to and potentially greater than adults.^40^ Recent studies have shed light on conditions of perinatal compromise that have direct effects on the NVU.^1^ Premature births are generally associated with a more fragile NVU and inflammatory response, increasing the risk of IVH.^41,42^ Acute hypoxia is commonly the result of hypoxic-ischaemic injury, associated with NVU changes such as astrocyte hypertrophy and hyperplasia in addition to disruption to the BM and TJ.^43^ Chronic hypoxia is frequently associated with FGR and is the focus of this review. The pathophysiology of brain injury in the FGR brain is complex, multicellular, and is likely mediated by both chronic hypoxia and neuroinflammation.^44,45^ Several animal studies have been conducted to characterise the effects of FGR on the NVU (Table 1). Current literature investigating the effects of FGR on the NVU primarily focus on altered vascularity and BBB dysfunction, underlying NVU cellular changes and the role of neuroinflammation at the NVU.

**Table 1:** Studies investigating the effects of FGR on the NVU.

Authors	Experimental model	Outcome measured	Key findings
Castillo-Melendez et al. (2015)^47^	Lambs delivered naturally at term (~145 days) and euthanised 24 h later FGR induced via SUAL at ~105 days gestation	White matter blood vessel density and number EC proliferation Pericyte and astrocyte attachment (coverage of vessels) BBB permeability Microbleeds	Reduced vessel density and number Reduced EC proliferation Reduced pericyte coverage, astrocytic end-feet loss of contact Increased BBB permeability—albumin extravasation and microhaemorrhages
Castillo-Melendez et al. (2017)^44^	Lambs delivered naturally at term (~145 days) and euthanised 24 h later FGR induced via SUAL at ~105 days gestation	Vessel density, proliferation (VEGF) EC proliferation Glut1 Pericyte and astrocyte coverage BBB permeability Microhaemorrhages	Reduced vessel density, proliferation (reduced VEGF expression) Reduced EC proliferation (reduced Glut1 expression) Reduced pericyte and astrocyte end-feet coverage Increased BBB permeability—albumin extravasation and microhaemorrhages
Chand et al. (2021)^49^	Term FGR piglets (<10th percentile birth weight) and NG piglets (10–90th) percentile Euthanasia on postnatal day 4	Vessel density, vessel length BBB integrity Microglial morphology Pro-inflammatory cytokines Neuronal apoptosis	Reduced vessel density and vascularisation and decrease in vessel branch points FGR showed pro-inflammatory environment with enlarged microglial cell bodies and thickened retracted processes FGR showed significant reductions in neural cells and increased apoptosis
Chand et al. (2022)^48^	Term FGR piglets (<10th percentile birth weight) and NG piglets (10–90th percentile) Euthanasia on postnatal day 4	Vascular integrity, density Juxtavascular Glial and astrocyte morphology BBB integrity Pro-inflammatory cytokines Apoptosis markers Tight-junction protein ZO-1	Reduced EC proliferation (reduced endothelial progenitor cells) Altered vascular integrity due to perturbed NVU composition Increased juxtavascular glial activation, increased Iba-1 positive microglia, altered astrocyte end-feet contact Increased frequency of hypertrophic astrocytic end-feet corresponding to decreased astrocytic end-feet coverage of blood vessels Altered BBB permeability and integrity—albumin and IgG extravasation associated with loss of astrocyte end-feet contact with NVU Increased juxtavascular and parenchymal cleaved-caspase 3 labelling, indicated increased apoptosis of astrocytes in NVU BBB disruption—decreased ZO-1 vessel coverage [diffuse and disjointed pattern in FGR vs continuous in NG]
Giambrone et al. (2019)^53^	Sprague-Dawley rats with sham surgery (abdominal incision only) vs induced placental ischaemia (surgical RUPP) on gestational day 14 Fetal brains collected on E19	Microbleeds—marker of vascular function Neuroinflammation Microglial density and morphology BBB permeability—brain water content	Increased vascular permeability—increased microhaemorrhages in parenchyma Neuroinflammatory state—increased pro-inflammatory cytokines Decreased microglial density in SVZ BBB permeability—surprising no difference in brain water content + contrary negative association between microbleeds and water content
Malhotra et al. (2018)^55^	Lambs delivered at 125 days gestation FGR induced via SUAL at 88 days gestation (term, 150 days)	BBB integrity—RBC, inflammatory cell infiltrate Neuroinflammation—astrogliosis (GFAP-positive staining) BBB integrity—astrocyte barrier disruption, albumin extravasation	Mild RBC infiltration accompanied by perivascular infiltrates of inflammatory cells within white matter consistent with microbleeds; Axonal injury within white matter Similar astrocyte cell counts between FGR and AGA Reactive morphology of astrocytes in FGR compared to AGA Disrupted interaction of astrocyte end-feet with cerebral blood vessels in FGR Albumin extravasation in FGR
Malhotra et al. (2020)^45^	Twin lambs delivered (127 days), intubated and ventilated then euthanised 24 h later FGR induced at 88 days gestation via SUAL in one twin	Neuroinflammation—microglial cell activation EC distribution and numbers through quantifying Glut1 Pericyte coverage BBB function—albumin extravasation	Increased number of activated microglial cells Increased EC coverage Decreased co-localisation with pericyte coverage BBB dysfunction—increased albumin extravasation
Yawno et al. (2019)^54^	Lambs studied at 115d GA, 124d GA, and 1 day postnatal (term is ~147d GA); FGR induced via SUAL at 105d GA	BBB function—microbleeds and albumin extravasation Astrocyte end-feet attachment, astrocyte density Neuroinflammatory response—microglial cell number	BBB dysfunction—blue staining and microbleeds present BBB dysfunction—albumin extravasation observed in all regions of cerebellum, especially adjacent to blood vessels Decreased end-feet of astrocytes associated with blood vessels, decreased astrocyte density in 124d GA Pro-inflammatory state—increase in microglial cell numbers at 124d GA

*AGA* appropriate for gestational age, *BBB* blood–brain barrier, *E(19)* embryonic day (19), *EC* endothelial cell, *FGR* fetal growth restriction, *GA* gestational age, *GFAP* glial fibrillary acidic protein, *Glut1* glucose transporter 1, *Iba-1* ionised calcium-binding adaptor molecule 1, *NG* normally grown, *NVU* neurovascular unit, *RBC* red blood cell, *RUPP* reduction of uterine perfusion pressure, *SUAL* single umbilical artery ligation, *SVZ* subventricular zone, *VEGF* vascular endothelial growth factor, *ZO-1* zonula occludens-1.

### Reduced vascularity

FGR leads to reduced vascularity in both white matter and grey matter regions. This is represented by findings of decreased vascular density and number of vessels, which has been hypothesised to indicate a breakdown of the BM in the NVU.^46^

Reduction in vascular density was observed in white matter regions of FGR lambs, including subcortical white matter (SCWM), periventricular white matter (PVWM) and the subventricular zone (SVZ).^44,47^ Using a laminin antibody (BM marker), this study demonstrated a reduction in vascular density throughout the white matter of FGR lambs^44,47^Similarly, an FGR piglet study visualised vascular density through another BM marker, collagen IV, and found reductions in vascularity within FGR brains, including reductions in total vessel lengths and vessel branching.^48,49^

Several FGR animal studies have observed alterations to ECs at the NVU. A study in FGR lambs showed a significant decrease in the numbers of Glut1-positive EC cells in three white matter regions (SCWM, PVWM, and SVZ).^44^ Furthermore, a significant number of these Glut1-positive cells were undergoing apoptosis in the FGR brain. Similarly, using other markers to examine ECs (CD34 and CD31), reduced numbers were demonstrated in the FGR piglet brain on postnatal day 4.^48,49^ In contrast, an FGR lamb study showed no difference in Glut1-positive EC counts in the same regions as the previous FGR lamb study. When the authors examined the number of cells per brain volume (as FGR brains may be smaller), they reported a significant increase in the number of Glut1-positive cells only in the PVWM.

### Blood–brain barrier dysfunction

#### BBB permeability

The increase in BBB permeability due to reduced pericytes and astrocyte attachment may cause FGR newborns to be at increased risk of brain bleeding and protein leakage. A well-established method of assessing BBB permeability is through the detection of serum protein extravasation into the brain parenchyma.^50–52^ Multiple FGR animal studies demonstrate extravasation of endogenous serum albumin, which is associated with reduced pericyte coverage as well as loss of astrocytic end-feet contact due to altered cell morphology, resulting in disruption of the NVU.^44,45,47–49,53,54^ In the FGR piglet brain,^48^ blood vessels undergoing apoptosis showed extravasation of albumin into the perivascular space and parenchyma. In the same study, extravasation of IgG into the cortex and white matter was also observed in vessels exhibiting altered astrocyte interaction.^48^ The resultant dysfunction of the BBB permeability may be associated with increased risks and incidence of brain pathology such as haemorrhages and oedema. This risk increases significantly with mechanical ventilation. In an FGR preterm lamb ventilation study, unventilated FGR lambs displayed mild degrees of microbleeds and albumin extravasation into brain parenchyma, whereas ventilated FGR lambs showed more severe microbleeds and extravasation in the form of increased red blood cell and albumin infiltration into brain parenchyma and capillary wall degradation in addition to periventricular leukomalacia.^55^

Disruption of BBB integrity in FGR may result in an influx of immune cells into the brain. Evidence of T-cell infiltration in the brains of FGR piglets has been shown using the pan T-cell marker, CD3^+^.^48^ T cells are involved in regulating immune responses and repair processes and can normally be found in the vessel lumen. In newborn FGR piglets, these CD3^+^ cells were observed in the perivascular regions and parenchyma of the brain.^48^ T-cell infiltration is reported to promote neuroinflammation and cognitive decline dependent upon subtype.^56^ While the study in FGR pigs identified CD3+ T cells, it did not further characterise these into helper (CD4+) or cytotoxic (CD8+) subsets. Thus, it is unclear whether this infiltration was a rescue or neuropathological response to BBB disruption.

#### Cellular changes

Pericytes are multifunctional cells involved in BBB maintenance, angiogenesis, and maturation of blood vessels. Pericytes embed into the capillary wall and encircle the vessel wall with their process, sharing the BM with ECs to regulate these processes.^57,58^ Three FGR lamb studies examining pericyte coverage of blood vessels using alpha smooth muscle actin and desmin immunolabelling, report a significant reduction of these cells throughout the FGR white matter.^44,45,47^ As a result, brain microvasculature may have increased instability and vulnerability to pathologies such as haemorrhage and oedema.^59^ Border-associated macrophages (BAMs) may also play a role in altering NVU integrity. Recent study has shown BAMs can increase vessel permeability and contribute to neurological dysfunction in a model of ischaemic stroke;^60^ however, the role of these cells in large animal models of FGR have not been investigated.

TJs are integral proteins controlling the paracellular and transcellular permeability of molecules into the CNS.^61^ TJs comprise of occludins, junctional adhesion molecules and claudins. Many studies in adult disease states demonstrate loss or mutations in TJs are associated with BBB disruption.^48,62–64^ Claudins are considered to be the key protein in determining ‘tightness’ of the barrier,^65^ with claudin-3 and -5 being the most prominently expressed in brain ECs.^66–68^ Claudin-5 in particular, is recognised as the critical protein for this function^66^ with its loss resulting in size-dependent permeability and death within 10 h of birth.^63^ Few studies have examined TJs in the FGR brain. Altered BBB permeability has been reported in the FGR piglet, however no change in transmembrane TJs claudin-5 and occludin were observed at postnatal day 4.^48^ The study reported significantly reduced protein levels of cytosolic TJ protein zonula occludens-1 (ZO-1), with diffuse and disjointed labelling of ZO-1 observed in FGR brain compared with NG. Whether this loss is maintained is known; however, loss of ZO-1 may be compensated by ZO-2 to form TJ complexes and thus reduce exacerbation of BBB disruption.^69^

The maintenance of claudin-5 and occludin protein levels may explain why overt BBB disruption due to loss of NVU components is not observed in FGR brain. While relatively normal levels of TJ proteins are reported in the FGR piglet brain, redistribution of these proteins, with movement from the membrane to the cytoplasm, may result in reduced BBB integrity because of altered TJ protein interactions. Redistribution of TJ proteins has been observed in other neurological disease animal models.^70^ It is also likely that altered expression of one TJ protein can influence interactions between other key TJs.^71^ Therefore, subtle alterations to TJ proteins in the FGR brain suggest that the increased BBB permeability observed may be due to altered function and interactions rather than overt breakdown of the barrier. Whether this is a permanent reduction/alteration in FGR brain needs further investigation.

Adherens junction proteins form cell–cell interactions between ECs contributing to the maintenance of a stable BBB and subsequently influencing paracellular permeability of the BBB^72^. The main regulators of this process are the transmembrane cadherins, primarily VE-, E- and N-cadherins. Loss of VE-cadherin expression at ECs is reported following hypoxia, which resulted in increased BBB permeability.^73^ Degradation of VE-cadherin expression is associated with increased expression of matrix metalloproteinase (MMP-3/9). Targeting MMP-3/9 expression may be a therapeutic approach to enhance BBB integrity following hypoxia-associated insults. GAP junction (GJ) proteins also form cell–cell junctions, allowing intercellular communication via diffusion of small molecules such as glutamate and adenosine triphosphate (ATP) between adjacent cells.^74^ Through influencing the movement of these small molecules, GJs are proposed to alter intracellular communication following injury. Whether this is neuroprotective or propagates injury has not been discerned in preclinical models. Connexins, key hemichannels at the BBB, are expressed by ECs, pericytes and astrocytes contributing to gliovascular coupling.^75^ The contribution of these proteins to the evolution of brain injury in FGR has not been thoroughly investigated; however, altered expression, translation, and cellular turnover can influence their function following injury. In preclinical models of perinatal hypoxic-ischaemic injury, there is a reported increase in Connexin-43 expression within hours and lasting days after insult induction which is implicated with increased BBB permeability.^76–78^ The therapeutic studies described in this review did not examine the expression of adherent or gap junction proteins in their respective models. Thorough characterisation of these proteins will determine whether therapeutic targeting may be a suitable strategy to protect the growth-restricted brain.

In the FGR brain, astrocytic morphology changes with shortened and thickening of processes.^48,49^ In both the FGR piglet and lamb brain, the altered morphology of astrocytes resulted in hypertrophic end-feet projections and reduced astrocytic end-feet interaction with cerebral vasculature.^47,48,55^

Microglia play an essential role in brain development, including vascular and axonal maturation.^79^ Similar to astrocytes, microglia at the NVU also demonstrate a shift in morphology from resting states to activated states in FGR. While glial activation on its own is not an indicator of inflammation, this altered morphology was associated with increased expression of inflammatory mediators.^80^ An increase in the juxtavascular glial activation is observed in FGR piglet brains with greater numbers of Iba-1-positive microglia.^48^ This study also demonstrated a negative correlation between astrocytic vessel coverage and the number of activated juxtavascular microglial cells, underscoring the interconnected nature of NVU components within the brain.

Neuroinflammation represents a central process in many disorders of the brain, and is thought to be an important mediator of atypical brain development in the FGR newborn.^81^ Neuroinflammation is primarily driven by the release of pro-inflammatory cytokines by activated glial cells, microglia and astrocytes.^82^ Neuroinflammation of the brain parenchyma is associated with adverse effects such as oedema and haemorrhage in FGR animals.^45,48,49,53,54^ This is largely due to an upregulation in pro-inflammatory cytokines nuclear factor κB (NF-κΒ), tumour necrosis factor α (TNFα), and interleukin-1β (IL-1β) released by microglia and astrocytes.^45,48,49,53^

A study in piglets examined key mediators of neuroinflammation at the NVU in FGR. Juxtavascular microglia and astrocytes in the NVU showed strong labelling for pro-inflammatory cytokines NF-κΒ, TNFα and IL-1β.^48^ Other strongly expressed cytokines include CXCL10 (IP10) in the vasculature as well as CCL2 in neuronal cells and CCL3 in microglia.^48^ The increased expression of these pro-inflammatory cytokines is associated with the activation of microglia in the NVU.

It is apparent that many of the pathological effects of FGR on the NVU stem in part from inflammatory processes affecting the anatomy and physiology of the NVU, resulting in varying types of BBB dysfunction. Neuroinflammation of the NVU in FGR may be associated with adverse effects such as haemorrhages and protein extravasation and is correlated with disruptions to BBB integrity.^83^ This is thought to be due to reactive changes in astrocytic morphology caused by a pro-inflammatory state which disrupts the permeability of the BBB.^48^ Giambrone et al.^53^ showed cerebral tissue IL-6 levels, a marker of neuroinflammation, were positively correlated with the frequencies of microbleeds in the FGR rat brain. In FGR lambs, neuroinflammation (microglia), was observed only at 124 days gestational age (GA) in the FGR sheep brain and not 115 days GA or postnatal day 1.^54^ This suggests that the period of perinatal development when the NVU is most vulnerable to neuroinflammation and disruption is at around 124 days GA, which is roughly equivalent to 34 weeks in human development.^84^ This correlates with patterns seen in human infants, whereby preterm FGR infants born beyond 34 weeks had a greater risk of IVH compared to FGR infants born at 28 weeks.^85^

The most severe FGR infants are born preterm and are therefore likely to require some form of respiratory support. There is a strong link between ventilation of the preterm appropriately-grown infant and brain injury.^86^ Studies in preterm fetal sheep have examined whether the risk of ventilation-induced brain injury is heightened in FGR infants.^55,87^ These studies show that in the short term, FGR lambs are highly susceptible to brain injury associated with mechanical ventilation. At a cellular level, prematurity and FGR increase levels of inflammation and oxidative stress, leading to BBB disruption and increased cell death^55,87^ observed through increased IL-8 levels and DNA fragmentation in cells.^87^ Clinically, the alterations to the BBB’s structure and function increase its permeability, and are associated with higher risks of IVH in infants.^55^ An important consideration for those FGR infants born very preterm, is the complex interaction between FGR, prematurity and ventilation. Often known as double jeopardy, this puts further pressure on the already stressed FGR brain and NVU.^45,55^ Fortunately, this combination of problems is rare, as most FGR babies are born moderate-late premature age not necessitating a need for ventilation. However, options for FGR treatment need to factor in this when babies are born very or extremely premature.

## Treatment effects on the perinatal NVU in FGR

The multiple disruptions to the NVU in the FGR brain result in detrimental consequences on the developing brain, with neuroinflammation underlying many of the pathological effects. Hence targeting inflammation may be key to preserving the multicellular NVU and central to providing neuroprotection in FGR. Several treatments containing anti-inflammatory properties have been studied in large animal models of FGR (Table 2).

**Table 2:** Studies investigating the effects of treatments on the NVU in FGR.

Authors	Experimental model	Intervention trialled	Key findings
Castillo-Melendez et al. (2017)^44^	Lambs delivered naturally at term (~147 days) and euthanised 24 h later FGR induced via SUAL at ~105 days gestation	Antenatal treatment with either saline (placebo) or Melatonin infusion (0.1 mg/kg) started 4 h after SUAL surgery	No significant difference in blood vessel density and proliferation (VEGF expression) between FGR group and intervention group Improved endothelial cell proliferation (Glut1 expression) with melatonin intervention Normalisation of pericyte and astrocyte end-feet coverage with melatonin intervention Albumin extravasation and microhaemorrhage prevention in the treatment group
Chand et al. (2021)^49^	Term FGR piglets (<10th percentile birth weight) and NG piglets (10–90th) percentile Euthanasia on postnatal day 4	cECFC/MSC/sham treatment administered on postnatal day 1	cECFC treatment increased vessel density while MSC had no effect cECFC treatment restored total vascular length as well as partially improved vessel branching cECFC reduced incidences of albumin and IgG-labelled area cECFC increased GFAP-positive vessel coverage cECFC microglial morphology similar to NG cECFC decreased glial activation and increased modulation of inflammatory mediators cECFC reduces neuronal apoptosis in the brain
Chand et al. (2022)^48^	FGR piglets (<10th percentile birth weight) and NG piglets (10–90th percentile) Euthanasia on postnatal day 4	Liquid ibuprofen was given via an oral dose of 20 mg/kg/day on postnatal day 1 and 10 mg/kg/day on days 2 and 3	Intervention group displayed juxtavascular astrocyte and microglia resting morphology similar to that observed in NG group Ibuprofen reduced the frequency of hypertrophic astrocyte end-feet and normalised vessel coverage Ibuprofen reduced the number of activated juxtavascular microglia Ibuprofen reduced pro-inflammatory cytokines and increased anti-inflammatory mediator Albumin and IgG extravasation reduced in ibuprofen treatment compared to untreated FGR Increased apoptosis in FGR ameliorated following ibuprofen treatment Decreased ZO-1 vessel coverage not recovered by ibuprofen treatment
Malhotra et al. (2020)^45^	Twin lambs delivered (127 days), intubated and ventilated then euthanised 24 h later FGR induced at 88 days gestation via SUAL in one twin	Allogeneic umbilical cord blood mononuclear cells (25 million/kg) were suspended in 2–3 ml of sterile saline and given intravenously (via the umbilical vein) to preterm ventilated lambs at 1 h of life	UCBC therapy reduced the number of activated microglial cells UCBC therapy resulted in significantly more increased endothelial cell coverage compared to FGR UCBC therapy normalised co-localisation of pericyte coverage UCBC therapy decreased albumin extravasation into the brain parenchyma
Bell et al. (2023)^113^	Preterm lambs studied at 127 days gestation FGR induced via SUAL at ~88 days gestation	1 × 10^7^ ECFCs delivered intravenously to fetal lambs in utero at 113 days gestation	ECFC administration increased both vessel sizes and overall vascular density throughout grey and white matter regions of both AGA and FGR lambs ECFC administration increased vascular astrocyte coverage in the cortical grey matter and subcortical white matter of both AGA and FGR lambs ECFC administration increased VEGF expression in the cortical grey matter and subcortical white matter of both AGA and FGR lambs ECFC administration produced no significant difference in vascular pericyte coverage in the brains of either AGA or FGR lambs ECFC administration produced no significant difference in vascular GLUT1 expression in the brains of either AGA or FGR lambs

*cECFC* combined endothelial colony-forming cells, *FGR* fetal growth restriction, *GFAP* glial fibrillary acidic protein, *Glut1* glucose transporter 1, *MSC* mesenchymal stromal cells, *NG* normally grown, *SUAL* single umbilical artery ligation, *UCBC* umbilical cord blood cell, *VEGF* vascular endothelial growth factor, *ZO-1* zonula occludens-1.

### Antenatal melatonin treatment

Melatonin (MLT) is a hormone primarily secreted by the pineal gland, and is an effective antioxidant with cell membrane stabilising properties.^88^ MLT-based treatments have been extensively tested for neuroprotection in various animal models of cerebral ischaemia, haemorrhage, and acute hypoxia-ischaemia where it exhibits anti-apoptotic, anti-inflammatory, and antioxidant properties while also preserving the integrity of the BBB.^89–97^ Interestingly, in one study, melatonin therapy was associated with adverse effects on fetal weight.^98^

In FGR lambs, antenatal MLT (0.1 mg/kg) infused from 105 to 147 days gestation, improved neurological outcomes by reducing fetoplacental oxidative stress as well as cerebral white- and grey matter injury.^94^ Yet, at the NVU, varying response to antenatal MLT treatment using the same dosage was observed in the FGR lamb.^44^ No significant differences in laminin-positive blood vessel density, vascular endothelial growth factor (VEGF) immunoreactivity, and number of proliferating blood vessels were evident between untreated FGR lambs and FGR lambs treated with antenatal MLT.

However, examining the effect of MLT treatment on ECs showed a positive effect. MLT treatment showed significantly increased Glut1 immunoreactivity in FGR lambs receiving treatment compared with FGR lambs. Although Glut1 immunoreactivity was increased in FGR lambs receiving MLT treatment, they were still significantly reduced compared to control lambs, demonstrating a partial recovery of ECs due to MLT treatment.^44^ In addition, there was a significant reduction in the number of apoptotic blood vessels in white matter regions of FGR-treated lambs compared with FGR non-treated lambs.

In terms of BBB dysfunction, pericyte and astrocytic end-feed coverage of the blood vessels in all three areas of the brain studied were significantly improved in FGR lambs treated with antenatal MLT compared to untreated FGR lambs, resulting in improved structural stability of the blood vessels as well as BBB integrity.^44^ FGR lambs receiving antenatal MLT treatment rarely exhibited albumin extravasation, while none of the FGR lambs that received antenatal MLT treatment displayed microhaemorrhages compared to 5 out of 9 untreated FGR lambs.

### Postnatal umbilical cord blood cell treatment

Interest in stem cell therapy for perinatal neuroprotection has greatly increased in recent times, with a number of studies trialling umbilical cord blood cell (UCBC) therapy.^99–101^ UCBC’s act via anti-inflammatory and immuno-modulatory effects, are anti-apoptotic, and release neurotrophic growth factors to support the damaged and surrounding perinatal brain tissue.^102^ Preclinical studies suggest UCBC therapy could prevent or slow the progression of perinatal brain injury,^103–106^ as well as provide long-term improvements to behavioural outcomes.^104–107^

Allogeneic ovine UCBC treatment reduced levels of the pro-inflammatory cytokine TNFα and numbers of activated microglia, in the white matter of FGR lambs subject to early onset FGR.^45^ However, no significant reduction in astrogliosis nor levels of any other pro-inflammatory cytokine measured in the study was reported following treatment.

At the NVU, UCBC therapy led to changes in EC coverage in FGR lambs. While FGR resulted in increased EC coverage in lambs, UCBC therapy similarly also increased EC coverage in both AGA and FGR lambs. As a result, FGR lambs receiving UCBC therapy showed significantly higher EC coverage compared to all other groups, while UCBC also increased vascular pericyte coverage in FGR lambs to levels equivalent to AGA. In addition, only 1 of the 6 FGR lambs administered UCBC therapy demonstrated albumin extravasation.

### Postnatal human endothelial colony-forming cells and mesenchymal stromal cells treatment

Endothelial colony-forming cells (ECFCs) are vascular progenitor cells found throughout the vasculature with extensive angiogenic potential.^108–110^ They can be isolated from many sources, but are typically taken from umbilical cord blood (UCB) and placenta.^111,112^ ECFCs have previously been studied in areas such as reperfusion of ischaemic tissue through angiogenesis.^110,112^

A recent investigation found ECFC treatment enhanced NVU development across several domains in both AGA and FGR fetal lambs.^113^ ECFCs used in this study were derived from human UCB and administered intravenously to preterm fetal lambs in utero, with results providing evidence to support the vasculogenic capacity of the cells in the perinatal brain. In particular, ECFC administration was associated with increased vascular density, an effect that was largely attributable to increased vessel sizes.^113^ This outcome was seen in both AGA and FGR preterm fetal lambs, occurring throughout each of the grey and white matter regions investigated. ECFC administration also led to increases in vascular astrocyte coverage and angiogenic signalling within cortical grey matter and SCWM, reinforcing the potential of ECFC-based treatments in enhancing perinatal NVU development and protection.^113^

It has been posited that the vasculogenic potential of ECFCs can be further amplified if co-administered with mesenchymal stromal cells (MSCs).^114^ Combined ECFC and MSC (cECFC) therapy has shown benefits such as the ability to bypass the host immune system^115^ as well as not requiring immunosuppressive therapy in immune-competent animals, thereby opening the possibilities for use as an allogeneic therapy.^110^

A recent study used the healthy human term placenta to extract fetally derived MSCs and ECFCs as a potential neuroprotective treatment in FGR piglets.^49^ Reduced vascularity observed in FGR in piglets was improved following cECFC treatment. Blood vessel density, vessel length, and vessel branching all showed significant increases in FGR piglets receiving cECFC treatment. In addition, decreases in the EC marker CD31 seen in the FGR brain were ameliorated following cECFC treatment. Of note, enhancements in vascular density were only observed in FGR piglets that received cECFC treatment; MSC treatment on its own did not produce any significant changes to vascular density.

In terms of BBB dysfunction, cECFC treatment reduced the incidence of extravasation of albumin and IgG in FGR piglet brains, although this incidence remained higher than appropriate for gestational-age piglets. However, GFAP-positive astrocytic vessel coverage in FGR piglets with cECFC treatment increased to levels similar to appropriate for gestational-age piglets, suggesting cECFC encourages the maturation of juxtavascular astrocytes.

cECFC treatment also had a modulating effect on neuroinflammation whereby microglial morphology in FGR-treated piglets more closely resembled a non-activated, resting state along with reduced astrocyte density. Yet, although MSC treatment alone reduced microglial activation, it had no significant effect on astrocytes. This suggests that while MSCs have anti-inflammatory effects, the combination cECFC is more effective.

### Postnatal ibuprofen treatment

As outlined above, neuroinflammation is a common effect of FGR, which has been shown to be associated with disruption to the NVU.^44,47^ Ibuprofen is a common non-steroidal anti-inflammatory drug that is currently used for the treatment of patent ductus arteriosus (PDA) in preterm neonates.^116^ A similar dosage (20 mg/kg day 1, and 10 mg/kg days 2 and 3) administered to FGR piglets has been shown to reduce inflammation and alleviate white and grey matter disruption.^117^

At the NVU, ibuprofen decreased neuroinflammation whereby the increased frequency of hypertrophic astrocyte end-feet and activated juxtavascular microglia in FGR piglets was ameliorated following treatment. The morphology of juxtavascular microglia and astrocytes in FGR ibuprofen-treated piglets was similar to NG piglets. Ibuprofen treatment also reduced inflammation in the FGR piglet brain parenchyma surrounding the NVU, with a reduction in pro-inflammatory cytokines (NF-κΒ, TNFα, IL-1β) as well as an increase in anti-inflammatory mediator IL-4.

Ibuprofen administration also reduced BBB disruption in the FGR piglet brain. A reduced frequency of IgG and albumin extravasation and improved preservation of astrocyte-vessel interaction were reported following treatment. TJs, a vital component of the NVU were also investigated and while FGR resulted in disruption to the cytosolic TJ ZO-1, ibuprofen treatment did not ameliorate this component of the BBB. It has been proposed that loss in ZO-1 and TJ redistribution is associated with increased IL-1b and/or VEGF derived from astrocytes and microglia.^118^ In epithelial cells, loss of cyclooxygenase-2 (COX2) was associated with the downregulation of ZO-1 which altered BBB permeability.^119^ It is plausible that expression of endothelial ZO-1 may be supressed by the non-selective inhibition of COX2 following ibuprofen administration.

Ibuprofen treatment also reduced T-cell infiltration into the FGR brain. Ibuprofen-treated FGR piglets displayed reduced levels of CD3^+^ T cells localised to perivascular regions and brain parenchyma with the majority localised at the vessel lumen, similar to NG piglets. Ibuprofen treatment also significantly reduced astrocytic Claudin-1 (Cldn1) labelling, a marker associated with CD3^+^ cell infiltration, by 28.6% in ibuprofen-treated piglets compared with untreated FGR piglets.

## Discussion

Although it is established that the NVU is associated with neuropathology in the developing perinatal brain, the pathophysiology of brain injury due to perinatal insults such as FGR is complex and not well understood.^44,45^ In this review, we summarised the current understanding of the impact of FGR on the perinatal NVU, which stem from large animal models and can be broadly categorised into altered vascularity in the brain, and BBB dysfunction. This is thought to be driven by neuroinflammation, which may be associated with cellular changes in the NVU, resulting in pathological effects in the FGR brain.^120^ Chand et al.^48,49^ demonstrated increased glial activation specifically at the NVU, with activated microglia displaying elevated expression of inflammatory mediators. These mediators enhance BBB disruption contributing to the exacerbation of brain injury in FGR.^121,122^ It is however unknown whether inflammation triggers these early alterations in BBB permeability or is the consequence of a ‘leaky’ barrier. Studies have shown that early and persistent inflammatory responses are associated with white matter and neuronal injury, and increased glial activation in the FGR brain.^45,81,123^ The loss of myelin-producing oligodendrocytes is associated with white matter injury (WMI) in a number of FGR models.^124–126^ Boccazzi et al.^127^ demonstrated oligodendrocytes are capable of blocking their own differentiation as well as shaping microglial activation in response to inflammation which may, in turn, contribute to WMI observed in neonatal brain injury. In response to inflammation, glial cells display activated morphology and release pro-inflammatory cytokines capable of inducing injury to neurons.^81,117,128^ These findings demonstrate the key interplay between cellular components of the NVU and the necessity for tight regulation of inflammatory pathways to maintain an optimum brain environment.

At present, no treatments exist that target neuropathology associated with FGR in the clinical setting.^49^ Neuroinflammation underpins many of the above-mentioned neuropathologies, providing a therapeutic target. A neuroprotective treatment to combat inflammation would either need to be given during pregnancy or after birth in the FGR setting. Yet, as mentioned below, there are challenges with both treatment timepoints.

**Fig. 2:**
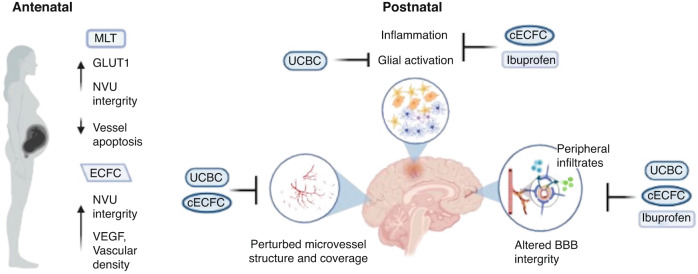
Summary of therapeutic treatment effects on the NVU in FGR. Melatonin (MLT) and endothelial colony-forming cells (ECFC) are administered antenatally, while umbilical cord blood cells (UCBC), combination ECFC and ibuprofen are administered postnatally. Each treatment response is unique, but all target the effects of FGR on the NVU studied thus far.

**Fig. 3:**
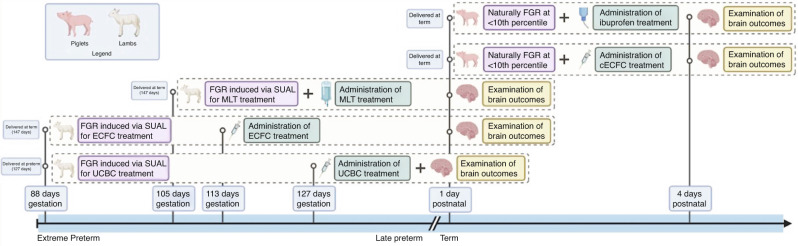
Timeline of therapeutic treatments in FGR. This timeline demonstrates the onset of FGR, timing of treatment administration, timing of delivery of animals, and the endpoint examination of brain outcomes.

Several FGR experimental models have tested various treatments with anti-inflammatory properties on the effects of FGR on the NVU (Table 2). Each intervention shows promise in FGR animal models and provides significant insight into the neuropathology of FGR as well as the treatment effects on the NVU (Fig. 2). However, further research is required to determine the optimum timing and dosing for each therapeutic. If these treatments are to be administered antenatally, they must cross the placenta and not adversely affect the mother and fetus. Treating postnatally may be easier from a delivery perspective, yet the injury has already been initiated. In the FGR piglet, both ibuprofen and placental stem cells were administered after birth.^48,49^ Demonstrating that even though the injury has been initiated, there is the potential for recovery in the postnatal period. Yet, treatment timing is an important consideration. Stem cell studies have shown administering cells at the height of an inflammatory insult can have no effect or may even worsen the injury.^129^ Each intervention reviewed here differed in their timing regarding the onset of FGR as well as of treatment provided (Fig. 3). The lamb trials exploring UCBC and ECFC treatment study early onset FGR,^45,113^ induced at 88 days gestation, which correlates to 25 weeks in human pregnancies while the lamb trial exploring MLT treatment induced FGR at 105 days gestation, which correlates closer to late-onset FGR in humans.^44^ It is worth noting that the piglets being trialled did not have surgically induced FGR, which means no data regarding the timing of FGR onset is available from piglets.^48,49^ The lamb trials examined brain outcomes on postnatal day 1, while the piglet trials looked at the NVU at postnatal day 4. Selecting a consistent time for the onset of FGR and therapeutic administration should be considered for future preclinical studies. The scope of this paper focused on the treatment of neuropathology in FGR, though the effects of these treatments on other important neonatal cardiovascular and respiratory morbidities also warrant further research.^45^

A further consideration for drug therapies is the role of ATP-binding cassette (ABC) transporters at the BBB in the clearance of drugs from the brain parenchyma into circulation. The primary ABC transporters include P-glycoproteins (P-gp), breast cancer resistance protein and multidrug resistance-associated proteins. The critical role of these proteins in protecting the fetal brain during development has recently been reviewed by Eng et al. (2022);^130^ however, these proteins have not been well characterised in neonatal FGR or other neonatal injuries such as hypoxic-ischaemic insults. Studies in adult models of hypoxic-ischaemic injuries demonstrate upregulation of P-gp,^131,132^ suggesting increased ability to clear xenobiotics from the brain subsequently reducing the efficacy of the administered therapy. Thus, further study is required to understand the roles and implications of ABC transporters in FGR brain injury and whether these can be targeted for therapies.

While the studies included in the current review have improved our understanding of the NVU and how it responds to FGR and various treatments, they are not without limitations. All studies investigated the short-term consequences of FGR on the NVU as well as short-term treatment responses. Further studies are required to examine the long-term impacts of FGR on the developing NVU as well as the long-term effectiveness and safety profile of promising treatments.

Multiple benefits of treatment have been identified in these studies, but further research is required to establish specific pathways and empirical causal relationships. For instance, due to histological constraints, it is not yet known if the positive effects of cECFC treatment on FGR piglets can be attributed to the functional improvements in the NVU or if it is a result of direct interaction between cECFCs and individual cells such as microglia and astrocytes.^49^ Although each trial treatment has demonstrated reductions in perivascular protein extravasation in the BBB, it is unclear if improvements in BBB function are directly tied to the anti-inflammatory effects of treatment.^48^ For instance, ibuprofen directly inhibits caspase catalysis independent of COX inhibition.^133^

While ibuprofen treatment appears promising due to its relatively low cost and established use for conditions such as PDA, the long-term safety profile has yet to be assessed in FGR neonates. The cell therapies investigated (cECFC, UCB, ECFC) consistently demonstrated enhancements of NVU components in FGR neonates compared with untreated cohorts. However, stem cell treatment may also result in neutral or negative effects as the cells may become entrapped in organs other than the brain when administered peripherally. Safety is paramount in this vulnerable FGR population and therefore cell type, timing and method of stem cell delivery need to be thoroughly investigated prior to translation to clinic.^134^ Understanding the impact of FGR on the developing NVU is essential to advancing the development of therapeutic treatments to improve outcomes in these vulnerable neonates.
